# Effects of antenatal corticosteroid therapy in animal models of fetal growth restriction: a systematic review and meta-analysis

**DOI:** 10.1186/s12884-025-07359-9

**Published:** 2025-03-13

**Authors:** Mette van de Meent, Dianne G. Kleuskens, Jan B. Derks, Floris Groenendaal, Wes Onland, Wessel Ganzevoort, A. Titia Lely, Carlijn R. Hooijmans, Judith Kooiman

**Affiliations:** 1 Department of Obstetrics, University Medical Center Utrecht, Wilhelmina Children’s Hospital, Lundlaan 6, Utrecht, EA 3584 The Netherlands; 2 Department of Neonatology, University Medical Center Utrecht, Wilhelmina Children’s Hospital, Lundlaan 6, Utrecht, EA 3584 The Netherlands; 3 Department of Neonatology, Amsterdam University Medical Center, Location AMC, Meibergdreef 9, Amsterdam, AZ 1105 The Netherlands; 4 Amsterdam Reproduction & Development Research Institute, Meibergdreef 9, Amsterdam, AZ 1105 The Netherlands; 5 Department of Obstetrics, Amsterdam University Medical Center, Location AMC, Meibergdreef 9, Amsterdam, AZ 1105 The Netherlands; 6 Department of Anesthesiology, Pain and Palliative Care, Meta Research Team, Radboud University Medical Center, Geert Grooteplein Zuid 10, Nijmegen, GA 6525 The Netherlands

**Keywords:** Systematic review, Meta-analysis, Animal models, Fetal growth restriction, Antenatal corticosteroid

## Abstract

**Background:**

Antenatal corticosteroids (CCS) reduce the risks of neonatal morbidity and mortality following spontaneous preterm birth. It is however debated whether effects of antenatal CCS hold for pregnancies complicated by fetal growth restriction (FGR) at risk for preterm birth. This study aimed to summarize effects of antenatal CCS in animal models of FGR by performing a systematic review and meta-analysis.

**Methods:**

The protocol for this systematic review was registered prospectively at PROSPERO (CRD42022318861). A systematic search was performed in PubMed and Embase from inception to April 5th 2023. Animal studies reporting on effects of antenatal CCS compared to placebo or non-treatment in FGR and appropriately grown offspring were included. Primary outcomes were fetal or neonatal mortality, organ developmental parameters (i.e. cardiac, brain, lung), glucose metabolism and fetal weight. Meta-analysis was performed using a random effects model. The quality of the included studies was assessed with the SYRCLE’s risk of bias tool.

**Results:**

The literature search yielded 10,386 unique hits. Eight studies were included in the systematic review. In terms of therapeutic effects, lung development and surfactant production were significantly accelerated by antenatal CCS in both FGR and non-FGR. Regarding drug safety, effects of antenatal CCS on fetal weight and brain development were similar for FGR and appropriately grown offspring except for one marker (4-hydroxynonenal) of brain injury, which was more pronounced in FGR offspring. Risk of bias appeared to be unclear for most studies across all domains.

**Conclusion:**

This systematic review illustrates that therapeutic and side effects of antenatal CCS are mainly similar in animal models of FGR as in non-FGR. These findings could therefore support the current recommendation of international guidelines to administer CCS to patients diagnosed with FGR at risk for preterm birth.

**Supplementary Information:**

The online version contains supplementary material available at 10.1186/s12884-025-07359-9.

## Background

Early-onset fetal growth restriction (FGR) is defined as failure of a fetus to meet its intrinsic growth potential, diagnosed before 32 weeks of gestational age. It is one of the major causes of stillbirth, neonatal morbidity and mortality and occurs in 0.5–1.0% of all pregnancies [1]. In developed countries, early-onset FGR is predominantly caused by placental dysfunction leading to unachieved nutritional and gaseous demands. To prevent stillbirth or severe neurological damage due to progressive hypoxia, early-onset FGR often requires iatrogenic preterm birth [2]. Also, early-onset FGR commonly coincides with pre-eclampsia, which can necessitate preterm birth as well [3].

Antenatal corticosteroids (CCS) reduce the risks of neonatal morbidity and mortality in imminent spontaneous preterm birth (< 34 weeks of gestation) [4]. The efficacy of antenatal CCS in the FGR population is however debated, especially when FGR is diagnosed before 32 weeks of pregnancy [5]. It is hypothesized that early-onset FGR fetuses are exposed to higher levels of endogenous glucocorticoids as a result of the more hypoxic intra-uterine environment [6]. Unfortunately, landmark trials on antenatal CCS in preterm birth did not include large numbers of early-onset FGR pregnancies, contributing to this gap in knowledge [4, 7]. Also, there are concerns about possible side-effects of CCS such as an increased risk of neurocognitive impairment, which could be even more pronounced in these already vulnerable fetuses [8, 9].

A randomized controlled trial comparing antenatal CCS with placebo in early-onset FGR pregnancies would provide the highest level of evidence on their efficacy. However, ethical considerations will make such a trial troublesome, if not impossible, as CCS administration is one of the very few antenatal strategies to improve neonatal outcome recommended in the guidelines [1, 10–12]. Another strategy to retrieve information about the possible therapeutic and side effects of CCS treatment is via animal models mimicking FGR. They can provide insight into the working mechanism of CCS in this specific field, and whether these effects differ between FGR and non-FGR pregnancies. The aim of this systematic review and meta-analysis is to summarize literature covering animal models of FGR comparing antenatal CCS with placebo on both efficacy and safety outcomes.

## Methods

The protocol for this systematic review was registered at PROSPERO (CRD42022318861) (Additional file 1), https://www.crd.york.ac.uk/prospero/) and reported according to the PRISMA Guidelines [13].

### Information sources and search strategy

On April 15th 2022 Pubmed and Embase were searched for studies describing effects of CCS treatment on offspring outcomes in animal models of FGR (updated 5 April 2023), which yielded 10,386 unique hits. The search string is provided in Additional file 2 [14]. No language or date restrictions were applied.

### Eligibility criteria

Studies were eligible if they reported on animal models of FGR comparing antenatal CCS with placebo or non-treatment. Outcomes of interest were all offspring outcome measures, including fetal and neonatal death, fetal and brain weight, organ developmental parameters (e.g. brain, lung, cardiac) and glucose metabolism. All animal FGR induction methods were included. Rayyan was used for screening and studies were excluded during title/abstract screening if (1) animals were non-mammals; (2) CCS treatment was combined with other intervention(s); (3) no correct control groups were reported; (4) the study was a human study, in vitro study, systematic review, book, conference abstract or case report [15]. Subsequently, studies were excluded during full text screening for abovementioned reasons and if (4) CCS were not administered correctly in terms of timing (e.g. postnatal) or route (e.g. oral administration); (5) FGR induction failed. Screening of retrieved literature was performed independently by two investigators (M.M. and D.K.). If no consensus was reached, a third investigator was consulted (J.K.).

### Data collection

Data regarding study characteristics were extracted, including species, FGR induction method, strain and fetal weights. Information was gathered about antenatal CCS administration dose, schedule, route and treatment duration. Mean, standard deviation (SD) and number (N) for both treatment group and control group were extracted for all outcomes for both FGR and non-FGR. Corresponding authors were contacted up to two times in case of missing data. Data were extracted by two independent investigators (M.M. and D.K.) with consultation of a third investigator (J.K.) if no consensus was reached.

### Assessment of risk of bias

Risk of bias was assessed by use of the SYRCLE’s risk of bias tool for animal studies, an adapted version of the Cochrane risk of bias tool, which has been used previously in multiple systematic reviews of animal studies [16–20]. In order to explain the expected large volume of studies with an unclear risk of bias judgement due to poor reporting of essential methodological details, three questions regarding the reporting quality were added (randomization, blinding and power calculation) [21]. This assessment was performed by two independent reviewers (M.M. and D.K.), discrepancies were resolved by consulting a third reviewer (C.H.).

### Data analysis

Outcome measures reported by at least three studies were described quantitatively by performing a meta-analysis using a random effects model, other outcome measures were described narratively. In order to assess the effect of antenatal CCS in FGR and non-FGR animals individual effect sizes (standardized mean differences (SMD) (Hedges’ g) were calculated for each comparison. Pooled difference were reported as SMD with corresponding 95% confidence intervals (CI). Subgroup analyses were planned for species if a subgroup contained at least ten independent comparisons. Between study heterogeneity was assessed by visual inspection of the forest plots and by use of the I^2^. Statistical analyses were performed in R-studio using the metafor package, version 4.0.3.1.32 [22].

### Publication bias

Publication bias analysis was planned if more than ten studies could be included in the meta-analysis by drafting a contour enhanced funnel plot and performing trim and fill analysis [23].

## Results

### Study selection and study characteristics

**Fig. 1:**
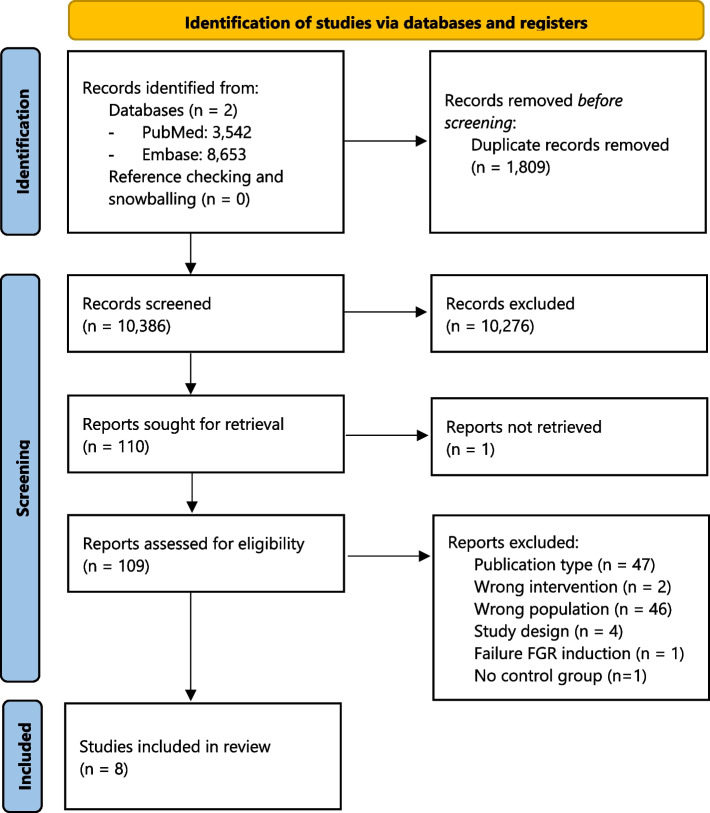
Flowchart of study selection. Abbreviation: FGR, fetal growth restriction

The literature search yielded 10,386 unique hits, of which eight studies met the inclusion criteria after full text screening (Fig. 1) [24–31]. The studies that were excluded after full-text screening are listed in Additional file 3. Five studies were performed in sheep, one in rats and two in guinea pigs. FGR was induced by single-umbilical artery ligation (*N* = 5), ablation of uterine artery branches (*N* = 1), uterine vessel ligation (*N* = 1) or starvation (*N* = 1). In six studies a single course of CCS was administered, i.e. two doses of CCS in 48 h, similar to the use of antenatal CCS in humans. The other two studies used repeated course of CCS from induction of FGR until delivery. All outcome measurements were performed within one day following CCS administration in all studies. Main characteristics of included studies are summarized in Table 1.

**Table 1:** Main characteristics of included studies

Author	Species	Strain	Model	Moment FGR induction (GA in days)	Label	N	Type of CCS	Dosage CCS	Dosage CCS (mg/kg)	Schedule CCS (GA in days)	Moment euthanasia (GA in days)	Term GA (days)	Fetal weight (grams)				Other reported outcomes
													Non-FGR – placebo	Non-FGR – CCS	FGR – placebo	FGR—CCS	
Lechner (1987) [24]	Guinea pig	Hartley	Starvation	45-term		140	Dexamethasone	2.0 mg	2	55-term, daily		70	104 ± 4	104 ± 5	69 ± 3ⁱⁱ	66 ± 3ⁱⁱ	Lung development, stillbirth
Manniello (1977) [25]	Rat	Sprague–Dawley	Uterine vessel ligation	17–19		528	9-α-fluoroprednisoIone acetate	1 mg/kg	1	24 and 48 h prior delivery	22	22	N/A	N/A	N/A	N/A	Glucose metabolism
McKendry (2010) [26]	Guinea pig	Tricolour	Ablation of uterine artery branches	30–35	Male Female	24 19	Betamethasone	1 mg/kg	1	61–65, daily	65	70	77.8 ± 4.22 78.56 ± 3.96	95.51 ± 4.82* 83.42 ± 10.63	53.72 ± 5.60ⁱ 52.12 ± 4.59ⁱ	67.78 ± 4.26 70.08 ± 5.73	Brain weight, brain development
Miller (2007) [28]	Sheep	Border-Leicester Merino	SUAL	108		18	Betamethasone	11.4 mg	0.15	113 and 114	115	154	2430 ± 90	1770 ± 70**	1690 ± 220**	1500 ± 150**	Brain weight, brain development
Miller (2012) [27]	Sheep		SUAL	108	Male Female	61 37	Betamethasone	11.4 mg	0.15	113 and 114	115	154	1880 ± 80 2080 ± 120	1670 ± 50* 1650 ± 80*	1530 ± 80* 1630 ± 120*	1450 ± 60* ⁱ 1280 ± 70* ⁱ	Brain weight
Sutherland (2012) [30]	Sheep	Border-Leicester Merino	SUAL	108		36	Betamethasone	11.4 mg	0.15	113 and 114	115	154	1990 ± 110	1640 ± 100*	1680 ± 110ⁱ 1680 ± 76ⁱ	1410 ± 110* ⁱ	Lung development, glucose metabolism
Sutherland (2020) [31]	Sheep	Border-Leicester Merino	SUAL	108	Short-term Long-term	34 28	Betamethasone	11.4 mg	0.15	113 and 114	115 125	154	1990 ± 110 3310 ± 200	1640 ± 100** 2530 ± 100**	1680 ± 110ⁱⁱ 2700 ± 150ⁱⁱ	1410 ± 100** ⁱⁱ 2160 ± 170** ⁱⁱ	Brain weight, brain development
Tare (2012) [29]	Sheep		SUAL	108		24	Betamethasone	11.4 mg	0.15	113 and 114	115	154	1939 ± 105	1723 ± 93	1473 ± 76ⁱ	1314 ± 89ⁱ	Heart

Values are presented as mean ± standard error

*Abbreviations*: *CCS* corticosteroids, *FGR* fetal growth restriction, *GA* gestational age, *kg* kilogram, *mg* milligram, *N/A* not applicable, *SUAL* single umbilical artery ligation

^*^P < 0.05 vs controls with placebo

ⁱ*P* < 0.05 non-FGR vs FGR

^**^P < 0.001 vs controls with placebo

ⁱⁱ*P* < 0.001 non-FGR vs FGR

### Synthesis of results

#### Therapeutic effects (Fig. 2)

**Fig. 2:**
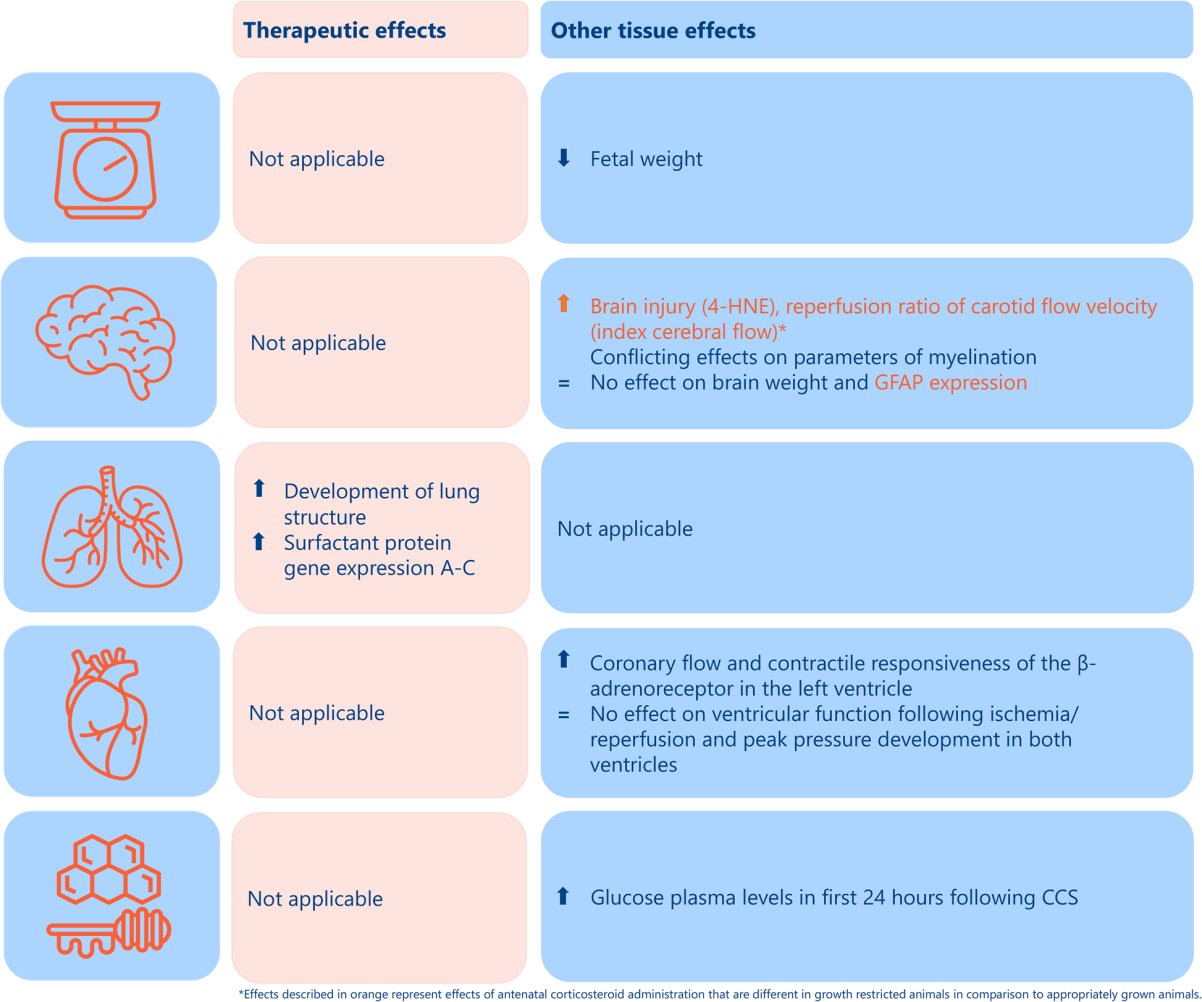
Effects of antenatal corticosteroid administration on weight, different organ structures and glucose levels in FGR sheep. Abbreviations: 4-HNE, 4-hydroxynonenal; CCS, corticosteroids; FGR, fetal growth restriction; GFAP, Glial Fibrillary Acidic Protein

##### Effects of antenatal CCS on lung development

Two studies described effects of antenatal CCS on lung development and surfactant production [24, 30].

In sheep, Sutherland et al., studied lung morphology by staining lung tissue sections with hematoxylin and eosin or Hart’s resorcin-fuschin to identify elastin and the presence of septal crests. Antenatal CCS stimulated lung development in a similar way in both FGR and appropriately grown offspring in comparison to controls receiving placebo, determined by a significant reduction in lung tissue density and Ki-67 positive cells (a monoclonal antibody for proliferating cells) (Fig. 2) [30, 32]. Also, surfactant protein gene expression was measured (quantitative real-time polymerase chain reaction), which significantly increased following antenatal CCS administration in both FGR and non-FGR (ratios of gene expression levels A-C in animals treated with CCS vs placebo varying between 2.5–12) (Fig. 2) [30]. Antenatal CCS did not alter the number of septal crests [30].

In guinea pigs, Lechner et al*.,* showed that antenatal CCS in both FGR and non-FGR significantly increased the alveolar epithelial surface area and the capillary endothelial surface area on light and electron microscopy in comparison to animals receiving saline. Also, CCS treatment increased the epithelial and endothelial tissue volumes and the alveolar surface density in FGR and non-FGR. The diffusing capacity for oxygen of the entire lung increased significantly in both FGR and non-FGR following CCS treatment compared to animals receiving placebo [24].

### Drug safety (Figs. 2, 3, and 4)

**Fig. 3:**
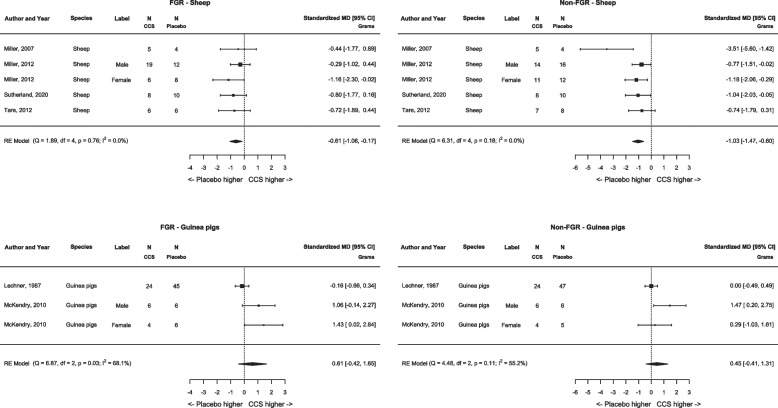
Meta-analysis on effect of antenatal corticosteroids on fetal weight per species. Forest plots show difference in fetal weight (grams) between antenatal corticosteroids and controls receiving placebo in fetal growth restricted offspring (left) and appropriately grown offspring (right). Data represent pooled estimates of standardized mean difference (SMD) with 95% confidence intervals (CI) using a random effect model. Abbreviations: CCS, corticosteroids; FGR, fetal growth restriction; RE, random-effects; MD, mean difference; I^2^, heterogeneity

**Fig. 4:**
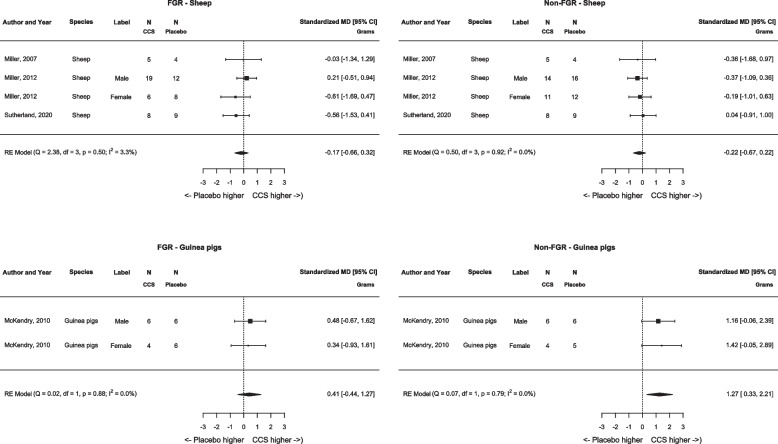
Meta-analysis on effect of antenatal corticosteroids on brain weight per species. Forest plots show difference in brain weight (grams) between antenatal corticosteroids and controls receiving placebo in fetal growth restricted offspring (left) and appropriately grown offspring (right). Data represent pooled estimates of standardized mean difference (SMD) with 95% confidence intervals (CI) using a random effect model. Abbreviations: CCS, corticosteroids; FGR, fetal growth restriction; RE, random-effects; MD, mean difference; I^2^, heterogeneity

#### Quantitative analyses

Initially, meta-analysis across all species was planned. However meta-analysis regarding fetal weight showed that the results were not robust across the various species, i.e. results in guinea pigs were contrary to the findings in sheep. A literature search based on this finding revealed that guinea pigs have glucocorticoid receptors with lower affinity rates for glucocorticoids [33–36]. The study of McKendry et al*.,* including guinea pigs, corrected for the lower affinity by administering a higher dose of CCS, it could however be questioned if this dose correction was sufficient (i.e. high enough). We therefore decided to conduct the analyses per species. The initial cross-species analyses are provided in Additional file 4–5.

#### Fetal and neonatal mortality

Lechner et al*.* showed that dexamethasone treatment significantly reduced the number of stillbirths in FGR guinea pigs (21% in starvation group vs 3% in the starvation group treated with dexamethasone), while this effect was not described in non-FGR. None of the included studies described neonatal mortality, as animals were euthanized during or after birth.

#### Fetal weight (Figs. 2, 3)

Six studies (eight comparisons) reported fetal weight comparing animals treated with CCS vs placebo (Table 1) [24, 26–29, 31]. The meta-analysis in sheep resulted in a significant reduction in fetal weight in both FGR and non-FGR (SMD −0.61 [95% CI, −1.06 to −0.17], *N* = 5, I^2^ = 0.0%; and SMD −1.03 [95% CI −1.47 to −0.60], *N* = 5, I^2^ = 0.0%, respectively) (Fig. 2, 3). The meta-analysis in guinea pigs showed no effect of CCS on fetal weight in both FGR and non-FGR (SMD 0.61 [95% CI, −0.42 to 1.65], *N* = 3 I^2^ = 68.1% and SMD 0.45 [95% CI −0.41 to 1.31], *N* = 3, I^2^ = 55.2%) (Fig. 3).

#### Brain (Figs. 2, 4)

Effects of antenatal CCS on the brain in FGR and non-FGR offspring were described in terms of brain weight, astrogliosis, expression of 5^α^-reductase type 1 and 2, myelination, brain injury and cerebral blood flow.

##### Brain weight

Four studies (six comparisons) desribed the effect of antenal CCS treatment on brain weight [26–28, 31]. In sheep, administration of antenatal CCS did not alter brain weight in FGR (SMD −0.04 [95% CI −0.59 to 0.51], *N* = 4, I^2^ = 0.0%), nor in non-FGR (SMD −0.30g [95% CI −0.80 to 0.20], *N* = 4, I^2^ = 0.0%) (Fig. 2, 4). Also, in FGR guinea pigs, antenatal CCS administration did not reduce brain weight (SMD 0.41 [95% CI −0.44 to 1.27], *N* = 2, I^2^ = 0.0%) (Fig. 4). However, in appropriately grown guinea pigs, antenatal CCS administration significantly increased brain weight (SMD 1.27 [95% CI 0.33 to 2.21], *N* = 2, I^2^ = 0.0%) (Fig. 4).

##### Astrogliosis

McKendry et al*.* and Sutherland et al*.* reported effects of antenatal CCS on glial fibrillary acidic protein (GFAP) immunoreactivity, a measure of astrogliosis in the fetal brain in both sheep and guinea pigs [26, 31]. This protein plays a major role in maintaining the integrity of the white matter of the central nervous system and the blood–brain barrier [26, 31, 37]. In both FGR sheep and guinea pigs, CCS treatment did not alter GFAP immunoreactivity (Fig. 2) [26, 31]. Nevertheless, antenatal CCS significantly decreased GFAP immunoreactivity in the corpus callosum of non-FGR sheep (Fig. 2) [31]. Also, in male guinea pigs, CCS administration significantly reduced GFAP expression in the CA1 region of the hippocampus [26].

##### Expression of 5^α^-reductase type 1 and 2

The 5^α^-reductase type 1 and 2 enzymes stimulate the conversion of progesterone to allopregnanolone. Allopregnanolone plays a major role in the development of the fetal brain and acts neuroprotective by reducing fetal brain injury following ischaemic insults [38]. In guinea pigs, expression of 5^α^-reductase type 1 and 2 in the brain was determined by real-time reverse transcriptase polymerase chain reaction [26]. Antenatal CCS increased expression of 5^α^-reductase type 1 similarly in both FGR and non-FGR female offspring only. However, a decrease of 5^α^-reductase type 2 was shown in male fetuses only following CCS in both FGR and non-FGR [26].

##### Myelination

In sheep, Sutherland et al*.* studied the effects of antenatal CCS on white matter brain development by measuring parameters of myelination (i.e. oligodendrocyte transcription factor 2 (Olig2), 2’−3’-cyclic-nucleotide 3’-phospodiesterase (CNPase), myelin basic protein (MBP)) [31]. Antenatal CCS administration increased CNPase in the external capsule in both FGR and non-FGR, while CCS reduced Olig2 in the subcortical white matter in both FGR and non-FGR (Fig. 2) [31]. Antenatal CCS did not influence other myelination parameters [31].

##### Brain injury

In sheep, Miller et al., described the effect of antenatal CCS on 4-hydroxynonenal (4-HNE), a marker of lipid peroxidation, and caspase-3 reactive cells, a marker for programmed cell death, collectively suggestive of brain injury [28]. CCS administration increased the number of 4-HNE reactive cells in both FGR and non-FGR offspring. Nevertheless, in the FGR group the number of 4-HNE reactive cells following antenatal CCS was significantly higher compared to the non-FGR receiving antenatal CCS, an effect which was not described in the FGR group treated with placebo (Fig. 2) [28]. CCS administration did not alter the number of caspase-3 reactive cells [28].

##### Cerebral blood flow

In sheep, Miller et al., determined the fetal carotid flow velocity as a proxy of cerebral blood flow at different time points following treatment with CCS. A reperfusion ratio was calculated by dividing the maximum carotid flow by the minimum carotid flow (preceding the maximum flow in the carotid) following CCS administration, which was significantly higher in FGR in comparison to non-FGR (1.91 [95% CI 1.68 to 2.28] and 1.50 [95% CI 1.35 to 1.85], respectively, *P* = 0.02) (Fig. 2) [28].

#### Heart (Fig. 2)

In sheep, Tare et al., studied the effects of antenatal CCS on coronary blood flow and maximal left and right ventricular pressure development by making use of the fetal Langendorff heart approach [29]. To add, contractile responsiveness was determined in both ventricles by administering an increasing dose of the β-adrenoreceptor agonist isoprenaline. Antenatal CCS administration increased coronary blood flow in both FGR and non-FGR [29]. CCS administration enhanced the contractile responsiveness of the β-adrenoreceptor in the left ventricle in both FGR and non-FGR (Fig. 2). Other cardiac outcome measures were not altered by CCS administration [29].

#### Glucose metabolism (Fig. 2)

In sheep, Sutherland et al., measured fetal glucose levels by collecting fetal arterial blood samples. During the first 24 h following CCS administration, levels of plasma glucose significantly increased compared to basal levels in both FGR and non-FGR to a similar extent (i.e. a more than threefold increase) (Fig. 2) [30].

In rats, Manniello et al. described plasma glucose levels during the first six hours after birth. Four to six hours after birth, plasma glucose levels of the FGR offspring treated with CCS levelled with glucose levels of appropriately grown offspring, which was not found in non-treated FGR offspring [25].

### Risk of bias of included studies

Risk of bias appeared to be unclear for most studies across all domains (Additional file 6). This was largely due to a lack of reporting sufficient information regarding adequate randomization and blinding.

### Publication bias

As a result of too few included studies, publication bias analyses were not conducted.

## Discussion

This systematic review and meta-analysis of eight animal studies suggest that the therapeutic effects of antenatal CCS regarding lung maturation and fetal mortality are as present in FGR as in non-FGR. In addition, the effects on fetal- and brain weight were similar in FGR and non-FGR. Importantly, except for the increase in 4-HNE (a marker of brain injury) and the reperfusion ratio of the carotid flow velocity, no additional neurological adverse effects of CCS were observed in FGR animals. Also, no adverse effects of CCS in FGR were reported for cardiac outcomes or glucose metabolism. Hence, in general, these findings would suggest that the efficacy and safety profile of antenatal CCS are similar in both FGR and non-FGR. These results could thereby support their use in the clinical setting of preterm birth in FGR.

Antenatal CCS have been extensively proven to be effective to accelerate lung maturation in pregnancies at risk for preterm birth in animal and human studies [4, 7, 39]. Nonetheless, their efficacy in FGR pregnancies has been questioned, as this population is being exposed to higher levels of endogenous glucocorticoids during pregnancy [40]. Subgroup analyses in pregnancies complicated by FGR have not been performed in the landmark trials studying the effects of antenatal CCS in preterm birth [4, 7]. Nevertheless, a meta-analysis on the effects of antenatal CCS in small-for-gestational age infants found a significant reduction in neonatal mortality [41]. The results of our systematic review and meta-analysis confirm the therapeutic effect of antenatal CCS in FGR, as antenatal CCS positively influenced lung development in FGR offspring of both sheep and guinea pigs [24, 30]. Regarding the safety profile of antenatal CCS, in both a clinical observational and randomized study, CCS negatively influenced fetal weight and head circumference in appropriately grown offspring, especially when multiple courses were administrated [42, 43]. In sheep, a significant reduction in fetal weight was found following antenatal CCS administration in both FGR and non-FGR, which underlines previously described effects in humans, while this effect was not observed in guinea pigs. This conflicting finding could be due to the lower affinity of the glucocorticoid receptor for glucocorticoids in guinea pigs compared to other species.

Conflicting results were found for the effects of antenatal CCS on fetal brain development. In both sheep and guinea pigs, no effect of antenatal CCS administration on GFAP expression was found in FGR [26, 31]. However, 5^α^-reductase type 2 expression in the brain was reduced in male FGR and non-FGR following CCS administration in guinea pigs. Male fetuses therefore might be more prone to adverse neurodevelopmental outcomes compared to female fetuses [26]. An observational study, which determined the number of developmental coordination disorders at eight years of age in humans, underlined this result [44]. With regard to white matter brain development, Sutherland et al*.* showed that administration of antenatal CCS increased white matter pathology in a similar way in both FGR and appropriately grown offspring [31]. Thus, the results of this study suggest that there is no treatment interaction between CCS administration and FGR on white matter brain development.

Miller et al*.* described higher rates of oxidative brain damage in FGR sheep following CCS administration compared to controls treated with CCS and FGR sheep treated with placebo. The authors explained this increase in oxidative damage by a higher rebound reperfusion rate of the carotid artery (serving as proxy of cerebral flow), which preceded an increase in 4-HNE reactive cells (linked to cell death), as described in other studies as well [28, 45, 46]. This rebound reperfusion is however not in line with studies describing fetal Doppler parameters following CCS administration in humans. Henry et al*.* summarized the effects of antenatal CCS administration on Doppler parameters in a systematic review and found that the pulsatility index of the middle cerebral artery showed no change in some studies and a transient reduction (i.e. increased cerebral flow) following CCS in other studies. Both of these findings are in contrast with the reduction in flow in the carotid artery, the proxy of cerebral flow, preceding the increased flow (and therefore a higher reperfusion rate) described by Miller et al*.* [47]. Nevertheless, considering the abovementioned effect of antenatal CCS on 4-HNE in the fetal brain, it can be postulated that antenatal CCS might negatively influence brain development, and possibly growth restricted fetuses are more prone to these negative effects. This concern was raised as well in a review article regarding the effects of CCS on brain development performed by Carson et al*.,* stating that the timing of neurodevelopmental development is altered by (earlier) exposure to synthetic glucocorticoids instead of maternal glucocorticoids (normally later in pregnancy) and that these effects might be more apparent in specific, vulnerable patient populations (e.g. FGR) [9].

Lastly, antenatal CCS and low fetal weight are both considered to be a risk factor for the development of neonatal hypoglycaemia [48]. Maniello et al. showed an increase in post-delivery glucose levels following CCS administration in both FGR and non-FGR [25]. Therefore, these results do not suggest that effects of antenatal CCS on neonatal glucose levels should negatively influence clinical decision making in FGR pregnancies.

Strengths of this systematic review comprise the extensive literature search which was performed in line with latest guidelines on systematic review and meta-analysis conductance [49]. In addition, all available FGR animal models were included. Nevertheless, this review has some limitations. First, the number of included studies was limited despite the extensive search, which reduces the certainty of evidence, but emphasizes that the literature on this clinically relevant topic is scarce. Further research in the form of an individual participant data meta-analysis including human FGR patients would provide more evidence on the effects of antenatal CCS in this population. Second, effects of FGR or antenatal CCS solely on study outcomes could not be determined, as this was beyond the scope of our literature search. Third, the risk of bias of the included studies was largely unclear due to poor reporting of essential methodological details, hampering the certainty in the presented evidence [16, 50, 51]. Fourth, this systematic review suffers from some indirectness, as there was no consistency in the results between species for all outcomes, hampering the external validity and translation to the clinical, human situation. Other factors further limiting the translation of results to the human situation include: (1) three studies were conducted in rodents and due to the shorter duration of gestation two or more doses of antenatal CCS encompass a relatively long treatment period during gestation compared to the human situation; (2) organ development occurs partly during the neonatal period in rodents, which would take place during the fetal period in the human situation; (3) certain animal models of FGR are less likely to mimic human placental insufficiency as they lack exposure to chronic hypoxia (e.g. starvation) which may affect fetal lung development as well (chronic hypoxia results in increased levels of endogenous steroids) [6, 52]. Fifth, the time intervals between CCS administration and outcome assessment were relatively short in the included studies and therefore, especially long-term effects, are difficult to assess. Future preclinical studies should therefore focus on both direct therapeutic and long-term safety outcomes (e.g. neurodevelopment), preferably in larger animal models of FGR as these most closely resemble the human situation of placental insufficiency, such as sheep. Finally, different types of CCS were administered in the included studies. Nevertheless, a systematic review comparing betamethasone versus dexamethasone on treatment effect did not show any significant differences [53].

## Conclusions

In conclusion, this study suggests that antenatal CCS administration in FGR animal models improves fetal lung maturation, with similar effects on fetal and brain weight in comparison to appropriately grown animals. This review therefore underlines the recommendations of international guidelines to administer antenatal CCS to FGR pregnancies at risk for preterm birth. Also, studies reporting on brain development yielded conflicting results in both FGR and non-FGR animals and outcomes of one study suggested that FGR offspring might be more prone to brain injury following CCS treatment in comparison to appropriately grown offspring. This topic should therefore be the main focus of future research.

## Supplementary Information

Additional file 1. PROSPERO registration.

Additional file 2. Search string Pubmed and Embase.

Additional file 3. List of excluded studies after full-text screening (*N*=101).

Additional file 4. Meta-analysis on effect of antenatal corticosteroids on fetal weight. Forest plots show difference in fetal weight (grams) between antenatal corticosteroids and controls receiving placebo in fetal growth restricted offspring (left) and appropriately grown offspring (right). Data represent pooled estimates of standardized mean difference (SMD) with 95% confidence intervals (CI) using a random effect model. *FGR induction by starvation (in other models FGR was induced by surgery). Abbreviations: CCS, corticosteroids; FGR, fetal growth restriction; RE, random-effects; MD, mean difference; I², heterogeneity.

Additional file 5. Meta-analysis on effect of antenatal corticosteroids on brain weight. Forest plots show difference in fetal weight (grams) between antenatal corticosteroids and controls receiving placebo in fetal growth restricted offspring (left) and appropriately grown offspring (right). Data represent pooled estimates of standardized mean difference (SMD) with 95% confidence intervals (CI) using a random effect model. Abbreviations: CCS, corticosteroids; FGR, fetal growth restriction; RE, random-effects; MD, mean difference; I², heterogeneity.

Additional file 6. Assessment of risk of bias and study quality.

## Data Availability

Data extracted from included studies can be requested for by consulting the corresponding author.

## Abbreviations

4-HNE: 4-Hydroxynonenal
CCS: Corticosteroids
CI: Confidence Interval
CNPase: 2′3’-Cyclic-Nucleotide 3’-Phosphodiesterase
FGR: Fetal Growth Restriction
GFAP: Glial Fibrillary Acidic Protein
MBP: Myelin Basic Protein
N: Number
Olig2: Oligodendrocyte transcription factor 2
Pdgfrα: Platelet Derived Growth Factor Receptor α chain monoclonal antibody
SD: Standard Deviation
SEM: Standard Error of the Mean
SMD: Standardized Mean Difference
TRUFFLE: Trial of Randomized Umbilical and Fetal Flow in Europe
