# Supplementary material for: Effects of antenatal corticosteroid therapy in animal models of fetal growth restriction: a systematic review and meta-analysis
Source: BMC Pregnancy Childbirth. 2025 Mar 13;25:281. doi: 10.1186/s12884-025-07359-9 (PMC11908052; doi:10.1186/s12884-025-07359-9)
Supplement: Supplementary file 1 — Additional file 1. PROSPERO registration. [file 12884_2025_7359_MOESM1_ESM.docx]

**Additional file 1.** PROSPERO registration

Animal review

Please select one of the options below to edit your record. Either option will create a new version of the record - the existing version will remain unchanged.

A list of fields that can be edited in an update can be found [here](https://www.crd.york.ac.uk/prospero/documents/Reduced%20field%20for%20updates.pdf)

# * Review title.

Give the working title of the review. This must be in English. The title should have the interventions or exposures being reviewed and the associated health or social problems.

The effects of antenatal administered corticosteroids in animal models of fetal growth restriction (FGR).

# Original language title.

For reviews in languages other than English, this field should be used to enter the title in the language of the review. This will be displayed together with the English language title.

# * Anticipated or actual start date.

Give the date when the systematic review commenced, or is expected to commence. 01/04/2022

# * Anticipated completion date.

Give the date by which the review is expected to be completed. 01/04/2024

# * Stage of review at time of this submission.

Indicate the stage of progress of the review by ticking the relevant Started and Completed boxes. Additional information may be added in the free text box provided.

Please note: Reviews that have progressed beyond the point of completing data extraction at the time of initial registration are not eligible for inclusion in PROSPERO. Should evidence of incorrect status and/or completion date being supplied at the time of submission come to light, the content of the PROSPERO record will be removed leaving only the title and named contact details and a statement that inaccuracies in the stage of the review date had been identified.

This field should be updated when any amendments are made to a published record and on completion and publication of the review.

The review has not yet started: No

## Review stage Started Completed

Preliminary searches Yes Yes

Piloting of the study selection process Yes Yes

Formal screening of search results against eligibility criteria Yes Yes

Data extraction Yes No

Risk of bias (quality) assessment No No

Review stage Started Completed

Data analysis No No

Provide any other relevant information about the stage of the review here.

The review has started: preliminary searches and piloting of the study selection process have been completed. Formal screening of search results against eligibility criteria has started (not able to change this using the tick boxes).

The review has started: preliminary searches and piloting of the study selection process have been completed. Formal screening of search results against eligibility criteria has started (not able to change this using the tick boxes).

# * Named contact.

The named contact acts as the guarantor for the accuracy of the information presented in the register record.

Mette van de Meent

Email salutation (e.g. "Dr Smith" or "Joanne") for correspondence:

Miss van de Meent

# * Named contact email.

Enter the electronic mail address of the named contact. [m.vandemeent@umcutrecht.nl](mailto:m.vandemeent@umcutrecht.nl)

# * Named contact address.

**PLEASE NOTE this information will be published in the PROSPERO record so please do not enter private information**

Enter the full postal address for the named contact. Lundlaan 6, 3584 EA Utrecht

# Named contact phone number

Enter the telephone number for the named contact, including international dialling code. 0031611714404

# * Organisational affiliation of the review.

Full title of the organisational affiliations for this review and website address if available. This field may be completed as ‘none’ if the review is not affiliated to any organisation.

UMC Utrecht

Organisation web address:

# * Review team members and their organisational affiliations.

Give the personal details and the organisational affiliations of each member of the review team. Affiliation refers to groups or organisations to which review team members belong. **NOTE: email and country are now mandatory fields for each person.**

Miss M. van de Meent. UMC Utrecht Dr J. Kooiman. UMC Utrecht

Assistant/Associate Professor A. T. Lely. UMC Utrecht Assistant/Associate Professor C. Hooijmans. Radboud UMC Miss Dianne Kleuskens. UMC Utrecht

# * Funding sources/sponsors

Give details of the individuals, organisations, groups or other legal entities who take responsibility for initiating, managing, sponsoring and/or financing the review. Any unique identification numbers assigned to the review by the individuals or bodies listed should be included.

ZonMW: Synthesis of evidence Grant number(s)

114024170

# * Conflicts of interest.

List any conditions that could lead to actual or perceived undue influence on judgements concerning the main topic investigated in the review.

None

# Collaborators.

Give the name, affiliation and role of any individuals or organisations who are working on the review but who are not listed as review team members.

# * Review question.

Give details of the question to be addressed by the review, clearly and precisely.

What are the effects of antenatal administered CCS on neonatal mortality, neonatal morbidity outcome measures and fetal weight in pregnancies complicated by FGR in animal models?

Context and rationale

Early FGR occurs in 1000-1500 pregnancies per year in the Netherlands. This is frequently caused by placental dysfunction, which leads to chronic hypoxemia. Therefore, early FGR is a notable cause of stillbirth, neonatal morbidity (24%) and neonatal mortality (8-19%). Preterm labour is initiated to prevent the fetus from stillbirth. The antenatal administration of corticosteroids (CCS) is widely used in pregnancies at risk for preterm birth, as it is associated with lower odds of neonatal morbidity and mortality in ‘normally grown’ fetuses. However, in pregnancies complicated by early FGR the effect of CCS is unknown.

Therefore, this study aims to answer the following research question: ‘What are the effects of antenatal administered CCS on neonatal morbidity, neonatal mortality outcome measures and fetal weight in pregnancies complicated by FGR in animal models?’ Main neonatal outcome measures are defined as birthweight, still birth, neonatal mortality and neonatal morbidity.

Furthermore, this systematic review aims to answer the following questions:

1. Differ the effects of antenatal CCS per animal or animal model.
2. Is there a dose-effect relation between the dose of CCS and the effects on neonatal outcome measures?
3. Is there a difference in main outcome effects between single course vs daily repeated CCS courses in FGR animals?
4. *** Searches.**

Give details of the sources to be searched, and any restrictions (e.g. language or publication period). The full search strategy is not required, but may be supplied as a link or attachment.

MEDLINE and EMBASE. In addition, reference lists of included studies and relevant research will be searched to identify other sources. No restrictions will be applied to the search.

# URL to search strategy.

Give a link to the search strategy or an example of a search strategy for a specific database if available (including the keywords that will be used in the search strategies).

[https://www.crd.york.ac.uk/PROSPEROFILES/318861_STRATEGY_20220620.pdf](http://www.crd.york.ac.uk/PROSPEROFILES/318861_STRATEGY_20220620.pdf) Do not make this file publicly available until the review is complete

# * Human disease modelled.

Give a short description of the disease, condition or healthcare domain being modelled. Pregnancies complicated by FGR.

# * Animals/population.

Give summary criteria for the animals being studied by the review, e.g. species, sex, details of disease model. Please include details of both inclusion and exclusion criteria.

Inclusion criteria:

Pregnant, animal models for FGR with a placenta.

Exclusion criteria:

Non-animals.

Non-pregnant.

No FGR.

# * Intervention(s), exposure(s).

Give full and clear descriptions of the nature of the interventions or the exposures to be reviewed (e.g. dosage, timing, frequency). Please include details of both inclusion and exclusion criteria.

Inclusion criteria:

Maternal, systemic (intravenous, intramuscular, subcutaneous) CCS administration during pregnancy.

Exclusion criteria:

Combined interventions (when not possible to deduct the effect of CCS alone). CCS administration before pregnancy or after birth.

# * Comparator(s)/control.

Where relevant, give details of the type(s) of control interventions against which the experimental condition(s) will be compared (e.g. another intervention or a non-exposed control group). Please include details of both inclusion and exclusion criteria.

Inclusion criteria:

No use of antenatal CCS administration: use of placebo, vehicle treated or untreated. Non-FGR animals: sham-treated or untreated.

Exclusion criteria:

No correct control group (FGR or CCS).

1. *** Study designs to be included.**

Give details of the study designs eligible for inclusion in the review. If there are no restrictions on the types of study design eligible for inclusion, or certain study types are excluded, this should be stated. Please include details of both inclusion and exclusion criteria.

Inclusion criteria:

Primary, peer reviewed animal studies.

Exclusion criteria:

Human studies. In vitro studies. Case reports.

# Other selection criteria or limitations applied.

Give details of any other inclusion and exclusion criteria, e.g. publication types (reviews, conference abstracts), publication date, or language restrictions.

Exclusion based on publication type:

Conference abstracts.

Books.

Review articles.

# * Outcome measure(s).

Give detail of the outcome measures to be considered for inclusion in the review. Please include details of both inclusion and exclusion criteria.

Inclusion criteria:

Neonatal mortality

Neonatal morbidity (lung- and brain development, cardiovascular development, glucose metabolism) Fetal weight

Fetal death

Exclusion criteria:

No report of main outcome measures.

# N/A.

This question does not apply to systematic reviews of animal studies for human health submissions.

# * Study selection and data extraction.

Procedure for study selection

First, pre-screening will be performed based on title/abstract. In this phase, studies will be selected based on the description of an animal model of FGR with administration of CCS.

Afterwards, full-text screening will be executed. In this phase, studies will be further examined for eligibility based on study outcomes.

Lastly, snowballing will be performed to identify any additional papers, using the reference lists of the full-text papers that have been included.

Two independent researchers (M.M. and D.K.) will screen the literature on title/abstract. Discrepancies will be discussed between the two researchers until consensus is reached. If no consensus is reached, decision will be made by a third researcher (J.K).

Prioritise the exclusion criteria Ti/ab phase:

1. Not an in vivo animal study (e.g. experimental animal models)
2. Not an original study
3. Non-pregnant animals used
4. No FGR
5. No CCS treatment
6. No control groups

Full-text phase:

See above +

1. Timing CCS treatment not correct (e.g. CCS after birth)
2. Route of administration CCS not correct
3. Not about predescribed outcome measures

Methods for data extraction

Two independent researchers (M.M. and D.K.) will retrieve the data from each article. Discrepancies will be resolved by a third researcher (J.K.). If results regarding the main outcome measures are not reported clearly, authors will be contacted (up to two times).

Data to be extracted: study design

Number of included animals, experimental vs observational design, duration of follow-up, method of outcome measurement

Data to be extracted: animal model Species.

Strain.

Model.

Timing and method of FGR induction. Litter size.

Numbers of stillbirth.

Data to be extracted: intervention of interest Route of administration.

Scheme of administration.

CCS used.

Dose (mg/kg/day).

Gestational days of CCS administered.

Data to be extracted: primary outcome(s)

Fetal or neonatal mortality: dichotomous, numbers (percentage).

Brain development: all relevant brain development outcome measures, continuous and dichotomous Lung development: all relevant lung development outcome measures, continuous and dichotomous Development other organ structures: all relevant outcome measures

Birthweight: continuous grams, mean or median with SD, IQR or range

Data to be extracted: secondary outcome(s) Not applicable.

Data to be extracted: other

Drop-outs and reason for drop-outs.

# * Risk of bias and/or quality assessment.

State whether and how risk of bias and/or study quality will be assessed. Assessment tools specific for pre-clinical animal studies include [SYRCLE’s risk of bias tool](https://bmcmedresmethodol.biomedcentral.com/articles/10.1186/1471-2288-14-43) and the [CAMARADES checklist](http://journals.sagepub.com/doi/abs/10.1038/sj.jcbfm.9600064?url_ver=Z39.88-2003&rfr_id=ori%3Arid%3Acrossref.org&rfr_dat=cr_pub%3Dpubmed) for study quality

No

No

Yes

No risk of bias and/or quality assessment planned

By use of SYRCLE’s risk of bias tool

By use of SYRCLE’s risk of bias tool adapted as follows:

added with three reporting questions: reporting of any randomization, any blinding and a power calculation.

By use of the CAMARADES checklist for study quality

No

By use of the CAMARADES checklist for study quality, adapted as follows:

No

Other criteria, namely

No

Method for risk of bias and/or quality assessment

Two independent researchers (M.M. and D.K.) will use the adapted SYRCLE’s Risk of Bias tool for animal studies. Any discrepancies will be resolved by a third reviewer (J.K.).

1. *** Strategy for data synthesis.**

Planned approach

The data regarding the aforementioned outcome measures will be combined and compared by performing a descriptive synthesis, we will extract whether or not the author described evidence for increase or decrease in the outcome measures. A meta-analysis does not seem feasible as the literature concerning this topic is limited, except for the outcome of birth weight and mortality.

Meta-analysis on the outcome of birth weight and/or mortality will be performed provided that there are at least 3 of more studies reporting this outcome for FGR animals, comparing antenatal CCS treatment with placebo.

Effect measure

Standardized mean difference in birth weight (as different species will be included) and risk ratio of mortality will be the outcomes of interest. Subgroup analyses will be performed, if feasible (N > 10) given the amount of included studies, on the subgroup species.

Effect models

Random effects model.

Heterogeneity

I² and visual inspection of the funnel plots.

Other

Not applicable.

# * Analysis of subgroups or subsets.

Subgroup analyses

Subgroup analyses will be performed, if feasible (N > 10) given the amount of included studies, on the subgroup species.

Sensitivity

Standardized mean difference vs mean difference birth weight in case only one species is used.

Publication bias

If more than 10 studies can be included on the outcome birth weight and/or mortality, a contour enhanced funnel plot will be drafted for visual assessment on the risk of publication bias. We will test for PB using trim and fill analysis.

# * Review type.

Type of review

Animal model review

Experimental animal exposure review Pre-clinical animal intervention review

No No Yes

# Language.

Select each country individually to add it to the list below, use the bin icon to remove any added in error.

English

There is not an English language summary

# * Country.

Select the country in which the review is being carried out from the drop down list. For multi-national collaborations select all the countries involved.

Netherlands

# Other registration details.

List other places where the systematic review protocol is registered. The name of the organisation and any unique identification number assigned to the review by that organisation should be included.

# Reference and/or URL for published protocol.

Give the citation and link for the published protocol, if there is one.

No I do not make this file publicly available until the review is complete

# Dissemination plans.

Give brief details of plans for communicating essential messages from the review to the appropriate audiences. No

# * Keywords.

Give words or phrases that best describe the review. Separate keywords with a semicolon or new line. Systematic review; fetal growth restriction; corticosteroids; animal models

# Details of any existing review of the same topic by the same authors.

Give details of earlier versions of the systematic review if an update of an existing review is being registered, including full bibliographic reference if possible.

# * Current review status.

Review status should be updated when the review is completed and when it is published.

Review_Ongoing

# Any additional information.

Provide any further information the review team consider relevant to the registration of the review.

# Details of final report/publication(s) or preprints if available.

This field should be left empty until details of the completed review are available OR you have a link to a preprint. Give the full citation for the preprint or final report or publication of the systematic review.
