# Supplementary material for: Effects of antenatal corticosteroid therapy in animal models of fetal growth restriction: a systematic review and meta-analysis
Source: BMC Pregnancy Childbirth. 2025 Mar 13;25:281. doi: 10.1186/s12884-025-07359-9 (PMC11908052; doi:10.1186/s12884-025-07359-9)
Supplement: Supplementary file 3 — Additional file 3. List of excluded studies after full-text screening (N=101). [file 12884_2025_7359_MOESM3_ESM.docx]

**Additional file 3.** List of excluded studies after full-text screening (N=101)

| **Number** | **Author, year** | **Title** | **Reason of exclusion** |
| --- | --- | --- | --- |
| 1 | Aagaard-Tillery, 2010 | Maternal administration of dexamethasone partially rescues pulmonary immaturity in a murine model of ERK3-/- mediated fetal growth restriction & neonatal lethality | Wrong publication type |
| 2 | Alex, 2013 | Activation of the sympathetic nervous system, is it key to the developmental origins of enhanced cardiovascular risk? | Wrong publication type |
| 3 | Alvarez, 1982 | [Liver glycogen, blood glucose, somatotropic hormone, and hypophyseal thyrotropin in neonatal rats treated with cortisol and in malnourished ones] | Wrong population |
| 4 | Angervall, 1969 | Overweight in offspring of cortisone-treated pregnant rats | Wrong population |
| 5 | Ballard, 1983 | Hormones and receptors in developing lung | Wrong publication type |
| 6 | Ballard, 1983 | Mode of action of glucocorticoids on lung maturation | Wrong publication type |
| 7 | Ballard, 1986 | Hormones and lung maturation | Wrong publication type |
| 8 | Belkacemi, 2011 | Altered placental development in undernourished rats: role of maternal glucocorticoids | Wrong intervention |
| 9 | Bordier, 2022 | Deglutition-related cardiorespiratory events | Wrong publication type |
| 10 | Botting, 2017 | Antenatal glucocorticoid therapy protects the chronically hypoxic fetus from programmed endothelial dysfunction and hypertension in adulthood | Wrong publication type |
| 11 | Brown, 1979 | Reversible induction of surfactant production in fetal lambs treated with glucocorticoid | Wrong publication type |
| 12 | Brumley, 1977 | Whole and disaturated lung phosphatidylcholine in cortisol treated, intrauterine growth retarded and twin control lambs at different gestational ages | Wrong intervention |
| 13 | Burkhardt, 2017 | Intrauterine Wachstumsretardierungals lebenslanges Risiko für das Kind | Wrong publication type |
| 14 | Cohen, 1973 | Plasma corticosterone concentration in the foetal rat | Wrong population |
| 15 | Csaba, 1967 | Effect of cortisone on the foetus of pregnant rats. An attempt at widening the concept of teratogenesis | Wrong population |
| 16 | Cuevas-Guaman, 2012 | Antenatal glucocorticoids and postnatal surfactant treatment partially rescues neonatal lethality and pulmonary immaturity in an ERK3-/-knockout (ko) murine model of intrauterine growth restriction (IUGR) | Wrong publication type |
| 17 | De Rensis, 2002 | Effect of prostaglandin and dexamethasone injection on farrowing and piglet neonatal growth | Wrong population |
| 18 | Doyle, 2022 | Antenatal corticosteroids and outcomes into adulthood | Wrong publication type |
| 19 | Ekelund, 1975 | Glucocorticoids and pulmonary maturity | Wrong publication type |
| 20 | Farrell, 1977 | Fetal lung development and the influence of glucocorticoids on pulmonary surfactant | Wrong publication type |
| 21 | Fee, 2022 | One percent of the clinical dose used for antenatal steroid therapy is sufficient to induce lung maturation when administered directly to the preterm ovine fetus | Wrong population |
| 22 | Funkhouser, 1977 | Glucocorticoids and fetal lung development | Wrong population |
| 23 | Georgiev, 1989 | The influence of betamethasone (celestone) on the fetal lung development in rats | Wrong population |
| 24 | Giannopoulos, 1982 | Heterogeneity and ontogenesis of progestin receptors in rabbit lung | Wrong population |
| 25 | Hallman, 1980 | Effects of betamethasone and ritodrine on phospholipids of alveolar lung lavage in premature rabbits | Wrong population |
| 26 | Hansson, 1966 | The parathyroids in corticosteroid-treated pregnant rats and their offspring. I. Effect of cortisone | Wrong population |
| 27 | Harris, 2011 | RNA sequencing of lung in an antenatal glucocorticoid-rescued pulmonary immaturity murine model reveals roles for Surfactant Protein B (SPB) and Corticotropin Releasing Hormone (CRH) | Wrong publication type |
| 28 | Hennington, 2013 | Fetal exposure to high levels of corticosterone in a low birth weight rat model | Wrong publication type |
| 29 | Hoffmann, 1976 | Free and conjugated steroids in maternal and fetal plasma in the cow near term | Wrong population |
| 30 | Jain, 2022 | Maternal Nutrition and Fetal/Infant Development | Wrong publication type |
| 31 | Jobe, 1990 | Lung development, surfactant and respiratory distress syndrome | Wrong publication type |
| 32 | Jobe, 2003 | Animal models of antenatal corticosteroids: Clinical implications | Wrong publication type |
| 33 | Jobe, 2019 | The Amazing Premature Lung | Wrong publication type |
| 34 | Johnson, 1984 | Glucocorticoids and the respiratory distress syndrome | Wrong publication type |
| 35 | Juanes, 1986 | Attenuation of postnatal hypoxia in the premature newborn rat by maternal treatment with dexamethasone: its relationship with lung phospholipid content | Wrong population |
| 36 | Jurgens, 1991 | The effect of dexamethasone on de novo synthesis and degradation of lecithin resp. reacylation of lysolecithin in the fetal rat lung and liver | Wrong population |
| 37 | Kaempf, 2017 | Antenatal corticosteroids for the late preterm infant and agnotology | Wrong publication type |
| 38 | Kendall, 1972 | The effect of dexamethasone on pregnancy in the rabbit | Wrong population |
| 39 | Kikkawa, 1971 | Morphologic development of fetal rabbit lung and its acceleration with cortisol | Wrong population |
| 40 | Kisanga, 2019 | Actions of glucocorticoids at the maternal-fetal interface | Wrong publication type |
| 41 | Klepac, 1982 | Influence of dexamethasone on growth and development of rat fetuses: Changes in nucleic acids and protein content | Wrong population |
| 42 | Kukushkina, 1984 | Effect on the fetus of various drugs used during pregnancy (experimental study) | Wrong population |
| 43 | Kukushkina, 1984 | The use of some drugs during pregnancy and their effect on the fetus (An experimental study) | Wrong population |
| 44 | Lacaze-Masmonteil, 1993 | Pulmonary surfactant proteins | Wrong publication type |
| 45 | Lai, 1981 | Rabbit fetal lung maturation altered by suppression of endogenous thyroid and corticosteroid hormones | Wrong publication type |
| 46 | Lopez Lopez, 1981 | Action of the betamimetic drugs and synthetized corticoids on the fetal pulmonary maturation, evaluated after the concentrations of total cortisol in the amniotic fluid | Wrong study design |
| 47 | Lugg, 1978 | The effect of dexamethasone on the activity of 11beta-hydroxysteroid dehydrogenase in the foetal rabbit lung during the final stage of gestation | Wrong population |
| 48 | Lukina, 1969 | Adverse effect of steroid hormone therapy during pregnancy on the embryo and fetus development | Wrong publication type |
| 49 | Mellis, 2022 | Late hydrocortisone does not prevent bronchopulmonary dysplasia | Wrong publication type |
| 50 | Miller, 1962 | Effect of cortisone on the developing mouse foetus | Wrong population |
| 51 | Miller, 2009 | Cardiovascular responses to maternal betamethasone administration in the intrauterine growth-restricted ovine fetus | No control group |
| 52 | Motoyama, 1971 | Effect of cortisol on the maturation of fetal rabbit lungs | Wrong population |
| 53 | Moya, 1992 | Effects of betamethasone and thyroid hormone on fetal rat lung maturation in vivo | Wrong population |
| 54 | Nanda, 2023 | Successful Management of Systemic Pseudohypoaldosteronism Type 1 in an Infant | Wrong study design |
| 55 | Nelson, 1976 | Effects of gestational age, dexamethasone, and metopirone on lecithin concentration in fetal lung tissue and amniotic fluid in rats and guinea pigs | Wrong population |
| 56 | Noorlander, 2006 | Ontogeny of hippocampal corticosteroid receptors: Effects of antenatal glucocorticoids in human and mouse | Wrong population |
| 57 | Nord, 1992 | Rat lung polychlorinated biphenyl-binding protein: Effect of glucocorticoids on the expression of the Clara cell-specific protein during fetal development | Wrong population |
| 58 | Ogi, 2023 | Biomarkers of Stress in Companion Animals | Wrong publication type |
| 59 | Oldenborg, 1977 | The enzymes of phosphatidylcholine biosynthesis in the fetal mouse lung. Effects of dexamethasone | Wrong population |
| 60 | Olson, 1979 | Role of glucocorticoids in lung maturation | Wrong publication type |
| 61 | Orgeig, 2010 | Does the intrauterine growth-restricted fetus benefit from antenatal glucocorticoids? | Wrong publication type |
| 62 | Parvez, 1976 | Foetal growth retardation and mortality by chronic dexamethasone administration to pregnant rats | Wrong population |
| 63 | Patterson, 1984 | Short term regulation of surfactant lipids in fetal lung | Wrong publication type |
| 64 | Pfannschmidt, 1983 | Glucocorticoid-dependent acceleration of maturation especially of the lungs, in incipient respiratory distress syndrome of newborn | Wrong population |
| 65 | Pfannschmidt, 1983 | [Glucocorticoid-induced acceleration of maturation, especially of the lung, in threatened respiratory distress syndrome of the premature infant] | Wrong population |
| 66 | Phelps, 1984 | Effect of dexamethasone on the synthesis of specific proteins in fetal rabbit lung in vivo and in organ culture | Wrong study design |
| 67 | Rogoyski, 1975 | Effect of hydrocortisone acetate on the development of mouse embryo and foetus | Full text not available |
| 68 | Saule, 1975 | [Anti-atelectasis factor (surfactant) and hyaline membrane disease in premature infants] | Wrong publication type |
| 69 | Seckl, 2000 | Antenatal glucocorticoid therapy: A caveat to the applause | Wrong publication type |
| 70 | Seckl, 1999 | Glucocorticoids and fetal programming | Wrong publication type |
| 71 | Sidhu, 2022 | Hypoglycemia and Hypernatremia in a Term Infant, Physiologic or Pathologic? | Wrong study design |
| 72 | Shim, 2023 | Does cord blood cortisol have a mediating effect on maternal prepregnancy body mass index and birth weight? | Wrong publication type |
| 73 | Shimizu, 1991 | Appearance of surfactant proteins, SP-A and SP-B, in developing rat lung and the effects of in vivo dexamethasone treatment | Wrong population |
| 74 | Siddik, 1979 | Lack of correlation between cortisol-induced precocious maturation of the fetal rabbit lung and drug metabolism | Wrong population |
| 75 | Sidhu, 1981 | Prescribing in pregnancy. Corticosteroids | Wrong publication type |
| 76 | Slotkin, 2018 | Does growth impairment underlie the adverse effects of dexamethasone on development of noradrenergic systems? | Wrong population |
| 77 | Socol, 1981 | Effect of corticosteroids on the lecithin/sphingomyelin ratio in the rhesus monkey | Wrong population |
| 78 | Soll, 1998 | Surfactant treatment of the very preterm infant | Wrong publication type |
| 79 | Stein, 1973 | Further data on the slit-lid mutant in mice and the effect of cortisone and doca on its development | Wrong population |
| 80 | Steinbeck, 1972 | Aspects of steroidal influence on fetal development | Wrong publication type |
| 81 | Stettner, 1995 | The effect of betamethasone on phosphatidylcholine species composition in fetal rat lungs | Wrong population |
| 82 | Stokes, 1982 | Effect of corticosteroids on postnatal lung function | Wrong publication type |
| 83 | Sutherland, 2010 | The pulmonary effects of betamethasone administration in the growth restricted ovine fetus | Wrong publication type |
| 84 | Sutherland, 2013 | What are the effects of melatonin administration in the growth restricted ovine fetus exposed to glucocorticoids? | Wrong publication type |
| 85 | Sutherland, 2012 | Protecting the growth restricted fetal brain following glucocorticoid administration | Wrong publication type |
| 86 | Szabo, 1982 | The effect of glucocorticoid treatment during pregnancy on lecithin synthesis in the fetal rat lung | Wrong population |
| 87 | Taeusch, 1972 | Accelerated lung maturation and increased survival in premature rabbits treated with hydrocortisone | Wrong population |
| 88 | Thakur, 2000 | Effect of dexamethasone on insulin-like growth factor-1 expression in a rabbit model of growth retardation | Wrong population |
| 89 | Thompson, 1974 | Fetal-maternal corticosteroid relationships in sheep during late pregnancy | Wrong population |
| 90 | Tongpob, 2019 | Measuring placental oxygenation in a rat model of intrauterine growth restriction (IUGR): Investigations with in-vivo magnetic resonance imaging (MRI) | Wrong publication type |
| 91 | Torday, 2004 | Does "A" stand for alveolization? | Wrong publication type |
| 92 | Travianko, 1984 | [Hormonal stimulation of the maturation of the fetal pulmonary surfactant system] | Wrong population |
| 93 | Tsai, 1984 | Effect of prenatal dexamethasone on immunoreactive 6-ketoprostaglandin F1 alpha levels in fetal rat lungs | Wrong population |
| 94 | Tschanz, 1996 | Postnatal lung development and its impairment by glucocorticoids | Wrong publication type |
| 95 | Tsukasa, 2023 | Individual low dose betamethasone acetate therapy improves fetal lung maturation response with reduced HPA axis and growth suppression in a sheep pregnant model | Wrong publication type |
| 96 | Usuda, 2022 | Low-dose antenatal betamethasone treatment achieves preterm lung maturation equivalent to that of the World Health Organization dexamethasone regimen but with reduced endocrine disruption in a sheep model of pregnancy | Wrong population |
| 97 | Velayo, 2010 | Effects of antenatal steroid therapy on neurodevelopment in an IUGR mouse model | Failure FGR induction |
| 98 | Ward, 1982 | Postnatal pulmonary studies on the premature rabbit exposed in utero to betamethasone | Wrong publication type |
| 99 | Ward, 1983 | Postnatal increase in airway surfactant in the premature rabbit exposed in utero to betamethasone | Wrong population |
| 100 | Yawno, 2012 | Betamethasone decreases allopregnanolone synthesis and induces brain injury in fetal sheep | Wrong publication type |
| 101 | Yu, 2016 | Early and late effects of prenatal corticosteroid treatment on the microRNA profiles of lung tissue in rats | Wrong population |
