# Supplementary figures and images for: Effects of antenatal corticosteroid therapy in animal models of fetal growth restriction: a systematic review and meta-analysis
Source: BMC Pregnancy Childbirth. 2025 Mar 13;25:281. doi: 10.1186/s12884-025-07359-9 (PMC11908052; doi:10.1186/s12884-025-07359-9)

FGR

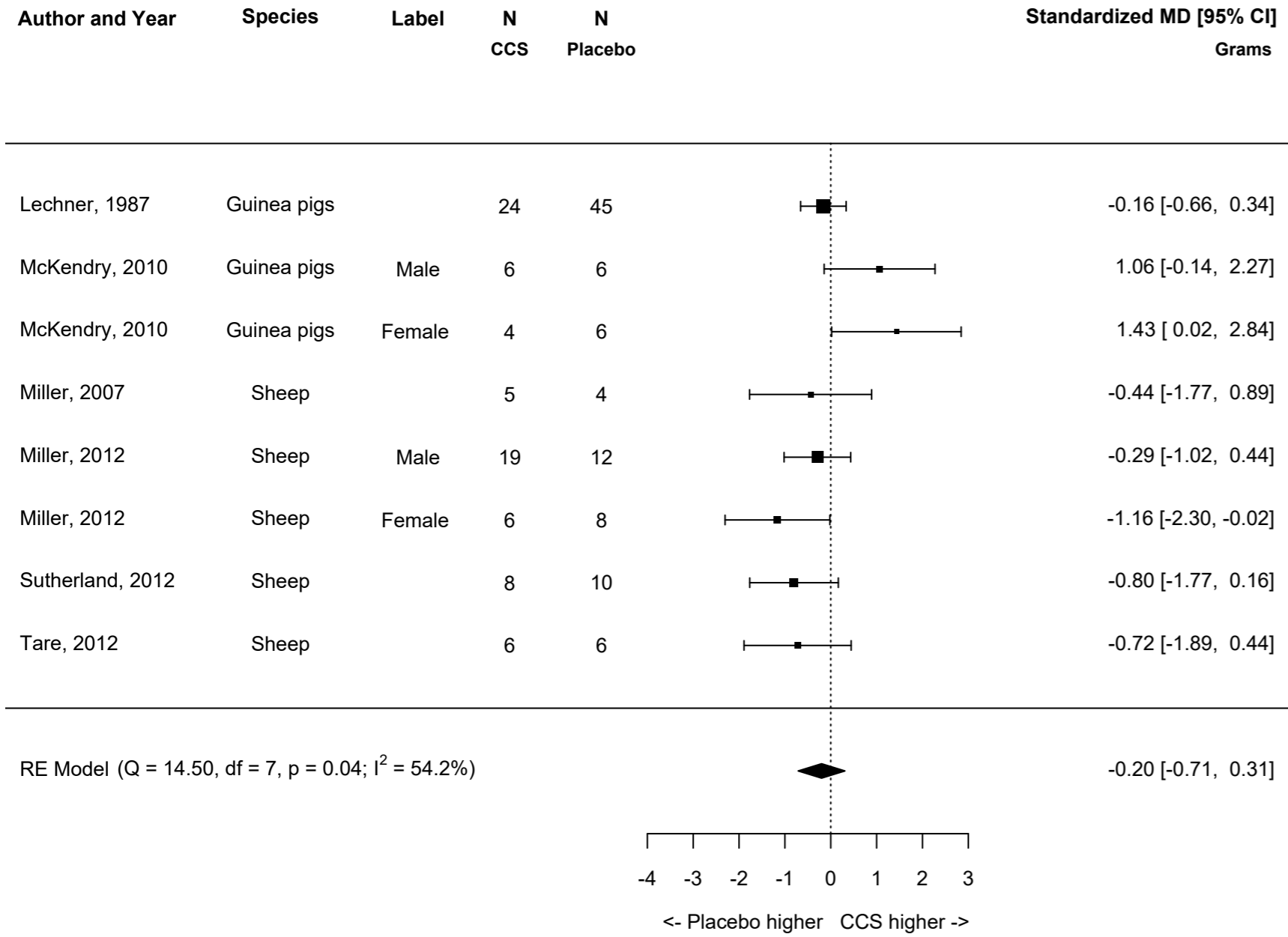

Non-FGR

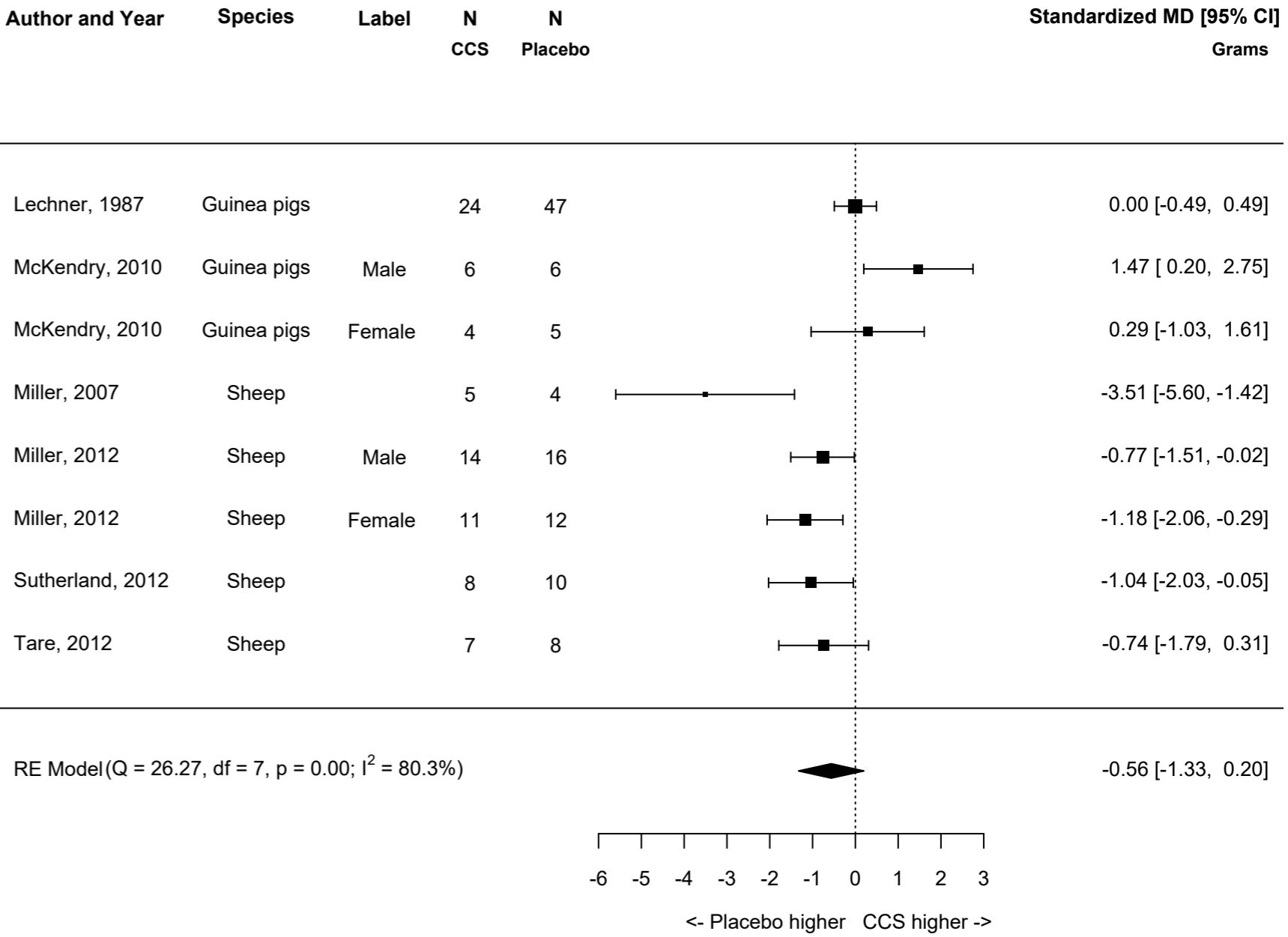

Supplement: Supplementary file 4 — Additional file 4. Meta-analysis on effect of antenatal corticosteroids on fetal weight. Forest plots show difference in fetal weight (grams) between antenatal corticosteroids and controls receiving placebo in fetal growth restricted offspring (left) and appropriately grown offspring (right). Data represent pooled estimates of standardized mean difference (SMD) with 95% confidence intervals (CI) using a random effect model. *FGR induction by starvation (in other models FGR was induced by surgery). Abbreviations: CCS, corticosteroids; FGR, fetal growth restriction; RE, random-effects; MD, mean difference; I², heterogeneity. [file 12884_2025_7359_MOESM4_ESM.pdf]

FGR

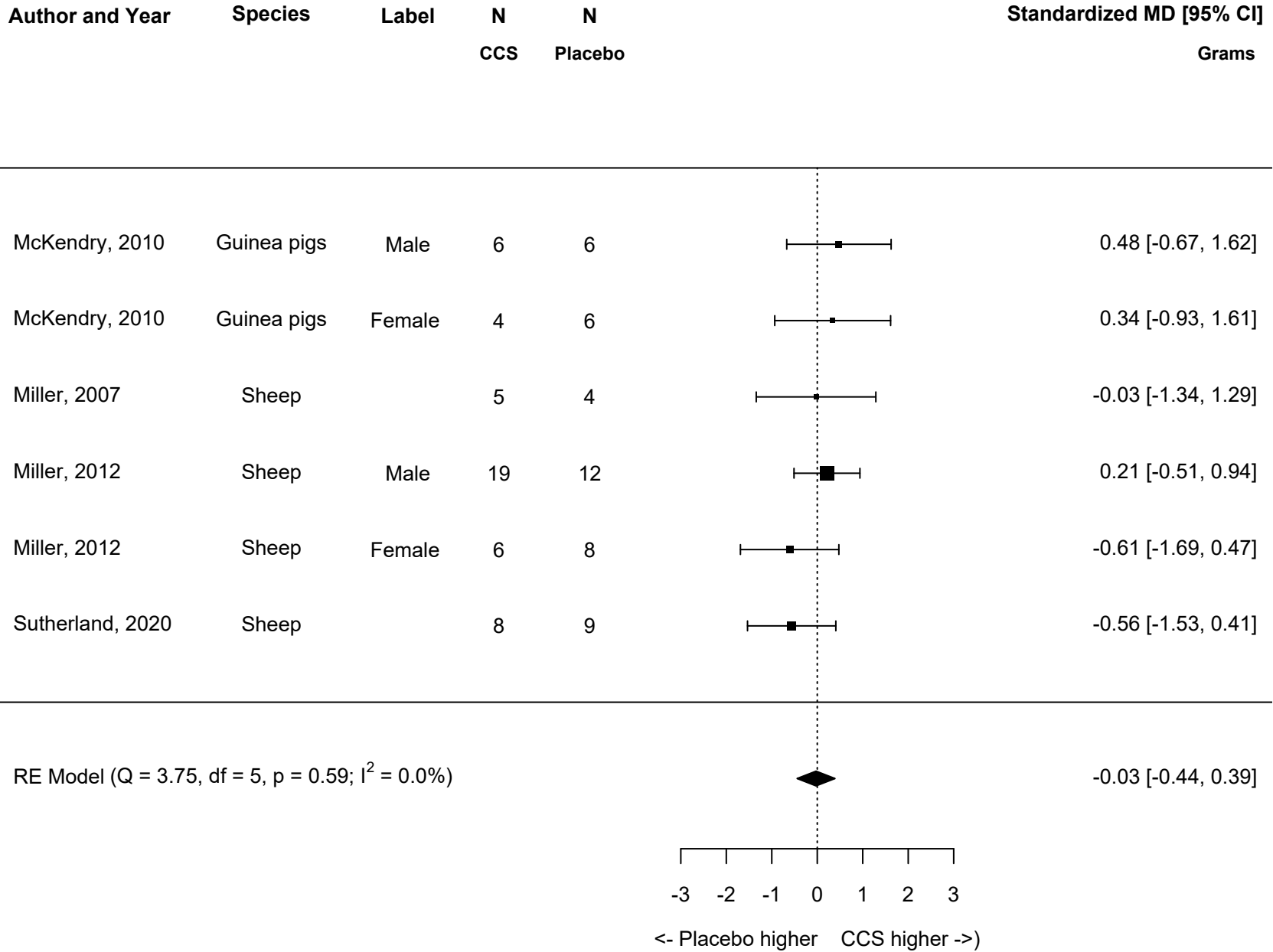

Non-FGR

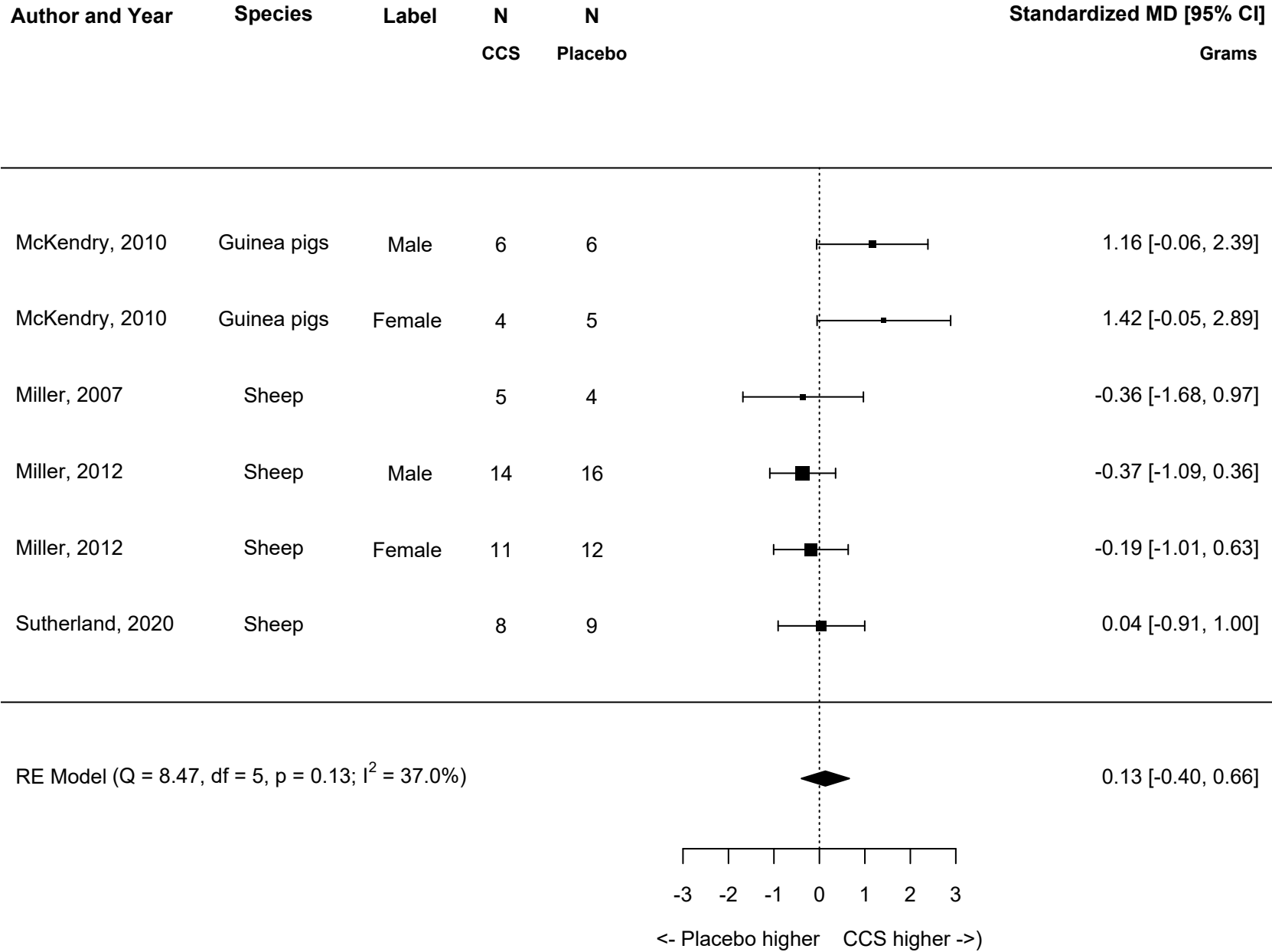

Supplement: Supplementary file 5 — Additional file 5. Meta-analysis on effect of antenatal corticosteroids on brain weight. Forest plots show difference in fetal weight (grams) between antenatal corticosteroids and controls receiving placebo in fetal growth restricted offspring (left) and appropriately grown offspring (right). Data represent pooled estimates of standardized mean difference (SMD) with 95% confidence intervals (CI) using a random effect model. Abbreviations: CCS, corticosteroids; FGR, fetal growth restriction; RE, random-effects; MD, mean difference; I², heterogeneity. [file 12884_2025_7359_MOESM5_ESM.pdf]
