# Supplementary material for: Effects of antenatal corticosteroid therapy in animal models of fetal growth restriction: a systematic review and meta-analysis
Source: BMC Pregnancy Childbirth. 2025 Mar 13;25:281. doi: 10.1186/s12884-025-07359-9 (PMC11908052; doi:10.1186/s12884-025-07359-9)
Supplement: Supplementary file 6 — Additional file 6. Assessment of risk of bias and study quality. [file 12884_2025_7359_MOESM6_ESM.docx]

**Additional file 6.** Assessment of Risk of Bias and Study Quality

|  | **Reporting** | | | | | **Risk of bias** | | | | | | | | | |
| --- | --- | --- | --- | --- | --- | --- | --- | --- | --- | --- | --- | --- | --- | --- | --- |
| **Author (year)** | Any randomization | Any blinding | Sample size calculation | Conflict of interest statement | Free of experimental unit of analysis errors | Random group allocation (selection) | Groups similar at baseline (selection | Blinded group allocation (selection) | Random housing (performance) | Blinded interventions (performance) | Random outcome assessment (detection) | Blinded outcome assessment (detection) | Reporting of drop-outs (attrition) | Selective outcome reporting (reporting) | Other biases |
| Lechner (1987) | Y | N | N | N | N | ? | ? | ? | ? | ? | ? | ? | ? | Y | ? |
| Manniello (1977) | N | Y | N | Y | N | ? | ? | ? | ? | ? | ? | Y | Y | Y | Y |
| McKendry (2010) | N | N | N | N | N | ? | ? | ? | ? | ? | ? | ? | ? | Y | ? |
| Miller (2007) | N | N | N | Y | N | ? | ? | ? | ? | ? | ? | ? | N | Y | Y |
| Miller (2012) | N | N | N | N | N | ? | Y | ? | ? | ? | ? | ? | ? | Y | ? |
| Sutherland (2012) | N | N | N | N | N | ? | ? | ? | ? | ? | ? | ? | ? | Y | ? |
| Sutherland (2020) | N | N | N | N | N | ? | ? | ? | ? | ? | ? | ? | Y | Y | ? |
| Tare (2012) | N | Y | N | N | N | ? | ? | ? | ? | ? | ? | Y | Y | Y | ? |
